# Term stillbirths in Eastern Uganda: a community-based prospective cohort study

**DOI:** 10.1080/16549716.2024.2448895

**Published:** 2025-02-03

**Authors:** Martin Chebet, Peter Olupot-Olupot, Andrew D Weeks, Ingunn Marie S Engebretsen, Noela Regina Akwi Okalany, Francis Okello, Thorkild Tylleskär, Kathy Burgoine, David Mukunya

**Affiliations:** a Department of Paediatrics and Child health, Busitema University, Mbale, Uganda; b Centre for International Health, Department of Global Public Health and Primary Care, University of Bergen, Bergen, Norway; c Department of Community and Public Health, Busitema University, Mbale, Uganda; d Department of Research, Mbale Clinical Research Institute, Mbale, Uganda; e Sanyu Research Unit, Department of Women’s and Children’s Health, University of Liverpool, Liverpool, UK; f Department of Paediatrics and Child Health, Mbale Regional Referral Hospital, Mbale, Uganda

**Keywords:** Stillbirths, term, causes, risk factors

## Abstract

**Background:**

Every year, 1.9 million stillbirths occur worldwide, of whom 1.5 million occur in sub-Saharan Africa (SSA) and Southeast Asia.

**Objectives:**

This study aims to determine the incidence and risk factors and to describe underlying causes for term stillbirths in Eastern Uganda.

**Methods:**

This was a cohort study of pregnant women enrolled at 34 weeks of gestation or more and followed to birth between January 2021 and January 2024. Enrolment and follow-up were done in the community by trained midwives. Using structured questionnaires, details of maternal health, pregnancy and birth were captured.

**Results:**

We enrolled 6101 participants and analysed 5496 for incidence of term stillbirth and 5296 for risk factors. Of the participants, 4913/5296 (92.8%) were between 14 and 35 years, and 4456/5296 (84.1%) had a health facility birth. There were 101 term stillbirths (61 were intrapartum and 40 antepartum). The incidence of term stillbirth was 18.4 per 1000 births (95% CI 14.8 to 22.9). The most common underlying causes of stillbirth were prolonged or obstructed labour 32/101 (31.7%) and malaria 20/101 (19.8%). The factors associated with term stillbirths were caesarean birth (aRR 3.3; 95% CI 2.00 to 5.4), intimate partner violence (aRR 1.8; 95% CI 1.1 to 2.8) and maternal age above 35 years (aRR 2.2; 95% CI 1.2 to 3.9).

**Conclusion:**

Eastern Uganda has a high rate of term stillbirths with more than half occurring during labour. Efforts are needed to improve the quality of birth care and to prevent intimate partner violence.

## Paper context

- **Main findings:** Prolonged labour and malaria were the most common attributable causes of term stillbirths in Eastern Uganda, while intimate partner violence and advanced maternal age were the identified risk factors.
- **Added knowledge:** Since there are limited recent data on stillbirths that include children born at home in Uganda, these results, which are based on over 6000 births, contribute to a better understanding of the current incidence, the common attributable causes and the risk factors of stillbirths in Uganda.
- **Global health impact for policy and action:** These results underscore the need for public health interventions to improve labour care, prevent malaria in pregnancy and reduce intimate partner violence in order to reduce stillbirths in Uganda.

## Background

Every year, nearly 2 million stillbirths occur worldwide, of whom 1.5 million occur in sub-Saharan Africa (SSA) and South East Asia [1,2]. To date, global attention to stillbirths has remained low. Stillbirths were absent in the Millennium Development Goals (MDGs) and remain absent in the Sustainable Development Goals (SDGs) [3,4]. Compared to the annual rate of reduction (ARR) of under-five and neonatal mortality, the ARR of stillbirths has been slower especially in the SSA. SSA has the highest rate of stillbirths and the slowest progress in reduction [5]. With the current rate of reduction, it is estimated that for a child born to a mother in SSA to have the same chance of being born alive as a child born to a mother in a high-income country (HIC) today, it will take another 160 years [5]. To accelerate reduction in stillbirths in SSA, more local research needs to be done to determine the burden and risk factors and identify potential strategies for prevention.

The risk factors for stillbirths can be classified according to the nature of the complication or its proximity to the causation of stillbirth. When classified according to their nature, they can be foetal, maternal, obstetric, delivery, health system, sociodemographic and lifestyle related, or unexplained/miscellaneous factors [6]. When classified according to proximity to causation of stillbirth, causative factors can be distant, intermediate or proximal [7,8]. The most common distant factors include lack of education in women, low socio-economic level and inability to make timely decisions [7–9]. The intermediate factors include maternal malnutrition, delay in seeking medical care, advanced or young maternal age and unavailability of community resources such as effective transport systems. Maternal medical conditions such as hypertensive disorders, infections and foetal factors such as congenital abnormalities and poor medical management of women are the proximal factors. In low-income countries (LICs), proximal causes including intrapartum-related complications, maternal medical conditions, congenital illnesses and infections are dominant, while intermediate and distant causes co-exist [10,11]. Previous studies have found that caesarean births, cephalo-pelvic disproportion leading to labour complications, prolonged or obstructed labour, premature rupture of membranes, hypertensive disorders in pregnancy, multiparity, second and third trimester bleeding, lack of antenatal care, congenital abnormalities, low birthweight, foetal distress, and cord accidents are associated with stillbirths [12]. Other risk factors include male sex of the infant, teenage pregnancies, advanced maternal age, smoking, and low maternal education [5,13,14].

Uganda’s stillbirth rate is estimated to be about 17 per 1000 total births [15]. Despite a high stillbirth burden in Uganda, there are limited data on the risk factors and probable causes of stillbirths. The common risk factors for stillbirths in Uganda identified in previous studies include maternal age 35 years or more, antepartum haemorrhage, malpresentation, prolonged/obstructed labour or other labour-related complications and distance from the nearest health facility among others [16,17].

Most of the published studies from Uganda are retrospective reviews, registry-based studies or are of older date [16,18–21]. There is a need for more recent and accurate primary studies to document the burden of stillbirths in Uganda.

Many term stillbirths are avoidable through early detection of risk factors at term, early delivery and appropriate management of labour [22]. It is important to understand the risk factors for term stillbirth in order to identify potential areas to focus on in future prevention strategies. The aim of this study was to determine the incidence and risk factors for term stillbirths among women in eastern Uganda.

## Methods

### Study design

This study was a prospective cohort study nested in the parent community cluster-randomized controlled trial, the BabyGel Trial, registered with Pan African Clinical Trial Registry: PACTR202004705649428, designed to evaluate the effectiveness of household alcohol-based hand rub (ABHR) for the prevention of sepsis, diarrhoea and pneumonia in Ugandan infants. The protocol is published [23].

### Study setting

This community study was conducted between January 2021 and January 2024 in Mbale and Budaka districts in Eastern Uganda. Mbale is located about 225 km northeast of Kampala, and Budaka district is located about 30 km west of Mbale. Both districts have predominantly rural populations.

### Inclusion and exclusion criteria

We included all women enrolled in the BabyGel trial [23]. In this trial, pregnant women of at least 34 weeks of gestation were enrolled in the community. The gestational age was determined based on the first trimester scan if available or the last normal menstrual period (LNMP) as per the antenatal care card or book. This card or book contained a summary of the findings at each antenatal visit the women had had. Women were expected to carry it for every antenatal visit as a reference for the health worker. Nearly all women who had at least one visit had the card or book. The LNMP was usually documented during the first antenatal visit. For almost all women, we used LNMP because they did not have a first trimester scan. If the LNMP or first trimester scan was not available, we used the fundal height recorded in the antenatal care card to determine the gestational age.

We included women above 18 years and those below 18 if they were emancipated minors. We only included women living in the participating villages who were intending to stay on for at least 3 months after the birth of their baby.

### Sampling and sample size

The number and size of the clusters were determined basing on the sample size requirements of the trial in which this study was nested, putting into consideration the planned duration of the trial. Based on the predetermined birth rates of the study area, to achieve a sample size of 5932 women over a two-year period, the trial needed to enrol women in 72 clusters each with a minimum population of 800.

Because the sample size for our study was limited by the size of the parent trial, we calculated the precision of the study. The sample size of 5496 that we analysed for the incidence of term stillbirth in this study resulted in an absolute precision of 0.4% to 1.3%, i.e. the difference between the point estimate and the 95% confidence interval (CI) for incidence values ranging from 2% to 50%. This precision is adequate for answering our primary objective.

To ease administration of the parent study and access to participants in the community, the study staff were located at three health centres (hubs), Budaka Health Centre IV in Budaka District, Busiu Health Centre IV and Nakaloke Health Centre III in Mbale. This was necessary because the study staff had to enrol and follow-up the participants in their homes.

We selected 24 villages within approximately 10 km radius from each health centre. The villages were selected based on their accessibility by data collectors on a motorcycle and proximity to one of the three health facilities. In each of the villages, all women who met the criteria were consecutively enrolled until the required sample size was reached.

### Study procedures

Women were approached in their homes and screened by trained research midwives. Informed consent was sought from those who were eligible. Women were enrolled from 34 weeks of gestation. A follow-up visit was done within 48 h after birth. During these visits, information concerning the mother’s health, pregnancy history, gynaecological history, socio-demographic and other information was captured in structured questionnaires. Questionnaires collecting baseline data antenatally and postnatally were developed for the BabyGel trial [23]. Among the sociodemographic variables, we captured the maternal age, educational level, marital status, wealth tertile, consumption of alcohol, intimate partner violence, partner’s level of education and partner’s consumption of alcohol among others. Wealth tertiles were calculated from a list of assets that women reported to possess, for example, radio, television, access to the internet and possession of a mobile phone, among others. We used this information to classify the participants into three groups: the richest, middle category and the poorest. Intimate partner violence was assessed by asking the women if they had forms of physical, sexual, emotional or psychological abuse from their partners. For clinical characteristics, we asked the women if they had a diagnosis by the health worker of malaria, urinary tract infection, hypertension, diabetes mellitus and others at any time during the pregnancy until birth. Women who reported fever that was not formally diagnosed by the health worker during pregnancy were classified as having ‘other fever’. We also captured the number of antenatal care visits until the time of delivery.

Previous pregnancy loss was assessed by asking the women if they had lost a pregnancy (miscarriage or stillbirth) before the current pregnancy. Biomedical distinction between abortion and stillbirth was difficult because these women could only report the approximate number of months the lost pregnancy was. Other clinical characteristics captured include type of delivery, sex of the child, HIV infection in the mother, birth place and number of previous births.

During the follow-up visit, we collected information from the mother about the circumstances surrounding the birth, any complications and her baby’s details. Determination of stillbirths and their causes was done by research midwives by interviewing the woman using a structured questionnaire developed for this study (supplementary file 1). This questionnaire was based on the World Health Organization (WHO) verbal autopsy tool for newborns, version 1.5.3 [24]. The WHO verbal autopsy tool for newborns has a section with questions dedicated to stillbirths. It has been validated for use in some LMICs [25] with high specificity and sensitivity. It has also previously been used to assess causes of stillbirths in different populations [26]. To triangulate the findings about stillbirth and its causes, we also reviewed the discharge form of the woman, inquired from the health worker who cared for the women at the health facility and reviewed health facility records about the baby’s birth. The discharge form given to the woman at the health facility after birth normally has a summary of the birth outcome. It was possible to interact with health workers and access most of the health records of the births because most of the women delivered from one of the three health facilities where the hubs were located. We had a medical officer affiliated with the study who was stationed at each of the three health facilities and acted as a link between the study staff and the health facility.

Stillbirth in this study was defined as the birth of a baby after an intra-uterine foetal death [27]. Babies born at least 37 completed weeks were considered to be born at term. Of these, those born dead were considered term stillbirths. The timing of the stillbirth was determined through the health facility record or the woman’s narrative. If this was not possible, if maceration was present, we classified the stillbirth as antepartum, while those without were classified as intrapartum. Presence of maceration was also determined from medical records or verbal reports by the women.

Concerning the underlying causes of stillbirth, the duration of labour was determined by the medical records, asking the health worker if this was possible or asking the women how long she spent in labour. Presence or absence of congenital abnormalities, antepartum haemorrhage, cord prolapse, maternal conditions such as hypertension and diabetes, and other causes was determined similarly. The woman was said to have had malaria, urinary tract infection or other illness if she had a diagnosis by the health worker during labour or at the time of diagnosis of foetal death in utero. Malaria diagnosis in these health facilities was usually done by malaria rapid diagnostic tests or microscopy. If there was more than one possible cause, these were designated as primary and secondary causes. The primary causes were then reported as the underlying cause of stillbirths

### Quality control

Data were collected using a structured questionnaire which was pretested in a language that the women were familiar with. The questionnaires were translated to the local languages (Luganda, Lugwere and Lumasaba) and back translated by professional translators. The data collectors were mostly midwives with diploma or bachelor’s degree who were trained to ensure quality data collection. Before the study started, they were trained about the protocol and data collection tools, among other trainings. The study processes, such as data collection, were closely monitored to detect any inefficiencies. Focused refresher trainings were done during the study when need arose. Completed questionnaires were checked for completeness at the end of every day. The study employed a quality assurance officer, a researcher and a PhD holder with a nursing and midwifery background who was trained in the protocol and had experience in monitoring clinical trials. The quality assurance officer ensured that the study procedures were done in accordance with the protocol through review of data collected and shadowing sampled data collection activities in the community among others.

### Data management and analysis

Data was collected electronically using REDCap software (Vanderbilt University) [28] in tablets and connected to internet daily for transmission to the server for storage. The server was based at the Clinical Trials Unit at Liverpool School of Tropical Medicine (LSTM). Data were analysed using Stata version 17.0 (StataCorp LLC, College Station, Texas, United States of America). Continuous variables were summarized as means with standard deviations or medians and interquartile ranges depending on the distribution. The categorical data were summarized as proportions. The incidence of term stillbirth was calculated from term stillbirths as a proportion of all term births and presented as term stillbirths per 1000 total births. The underlying causes of stillbirths were summarized using frequencies.

To assess factors associated with stillbirths, we used generalized estimation equations for the Poisson family, with a log link considering clustering and assuming an exchangeable correlation. Based on prior literature and expected incidence proportion of outcomes, we chose 11 factors to be included in the model for multivariable analysis of factors associated with term stillbirth.

### Ethics

The study was done in accordance with the Declaration of Helsinki. We obtained informed consent from all participants prior to enrolment. Ethical approval to conduct the study was obtained from the Research and Ethics Committee of the University of Liverpool (UoL004457), Regional Committee for Medical and Health Research Ethics, Norway (REK West #47004), CURE Hospital Uganda (CCHU-REC/21/019) and Uganda National Council of Science and Technology (UNCST, #HS919ES).

## Results

### Study population

**Figure 1.**
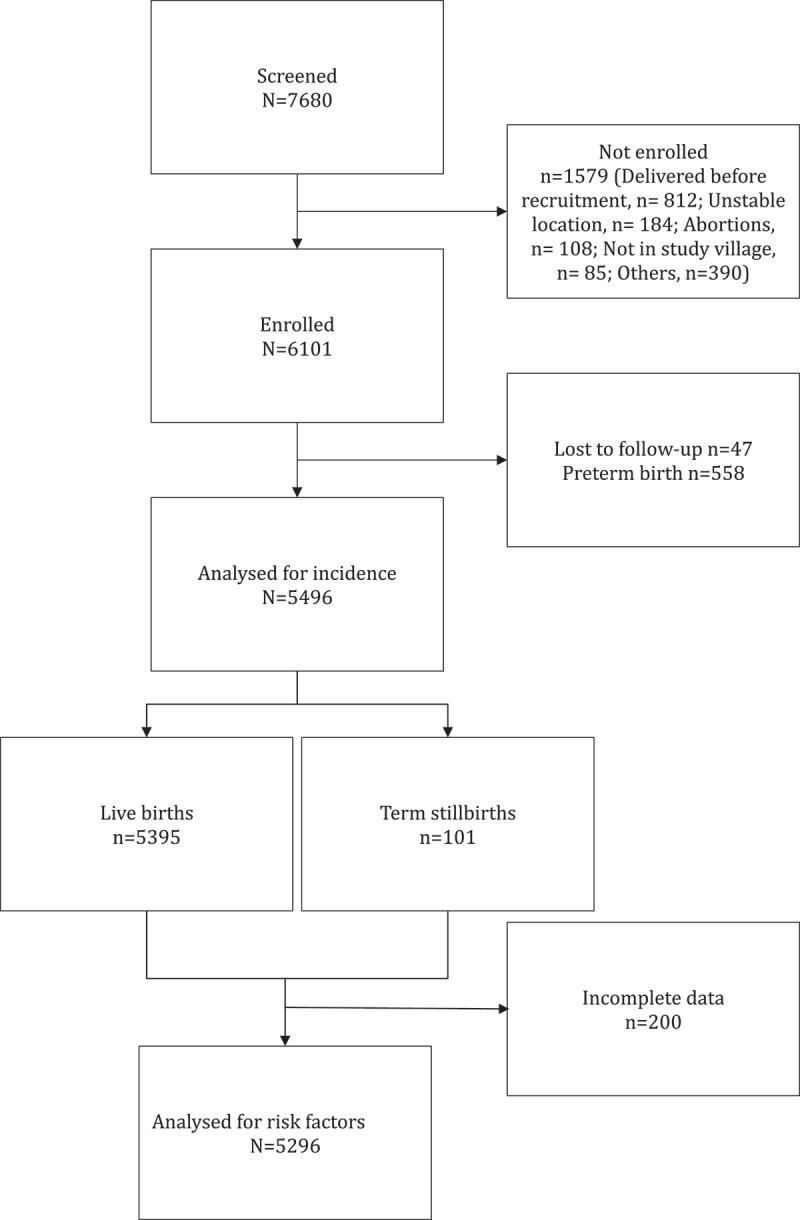
Study profile.

We screened 7680 pregnant women out of whom we enrolled 6101. A total of 1579 of the screened women were not enrolled, most of whom (*n* = 812) delivered before they could be enrolled because of COVID-19 lockdown restrictions against physical interactions. After screening them, there was a COVID-19 lock down which prohibited physical interaction. Research assistants were only able to make physical contact with these women after the restrictions were lifted. A total of 47 participants were lost to follow-up prior to birth and a further 558 gave birth before 37 weeks of gestation. We therefore analysed 5496 births for the incidence of term stillbirth. Since 200 participants had some missing data, we analysed 5296 births for factors associated with term stillbirths. Overall, 101 were term stillbirths (Figure 1).

About 92.8% (*n* = 4913) of the participants were between 14 and 35 years of age of whom, 19.6% (*n* = 1035) were below 20 years. The mean age of the women was 25.2 years [standard deviation (SD) 5.99] and 89.1% were either married or cohabiting, 35.5% had 8 or more years of formal education, 17.9% reported intimate partner violence and 10.0% reported drinking alcohol in pregnancy (Table 1).

**Table 1.** Sociodemographic characteristics of study participants (*N* = 5296).

	Live births N=5206 n (%)	Term stillbirths N=90 n (%)	Total N=5296 n (%)
**Maternal age (years)**			
14–35	4837 (92.9)	76 (84.4)	4913 (92.8)
Above 35	369 (7.1)	14 (15.6)	383 (7.2)
**Maternal education**			
Less than 8 years	3364 (64.6)	52 (57.8)	3416 (64.5)
8 or more years	1842 (35.4)	38 (42.2)	1880 (35.5)
**Marital status**			
Married/cohabiting	4645 (89.2)	75 (83.3)	4720 (89.1)
Single/divorced	550 (10.6)	15 (16.7)	565 (10.7)
Missing*	11 (0.2)	0 (0.0)	11 (0.2)
**Woman consumes alcohol**			
No	4684 (90.0)	82 (91.1)	4766 (90.0)
Yes	522 (10.0)	8 (8.9)	530 (10.0)
**Intimate partner violence**			
No	4285 (82.3)	65 (72.2)	4350 (82.1)
Yes	921 (17.7)	25 (27.8)	946 (17.9)
**Partner smokes**			
No	4768 (91.6)	85 (94.4)	4853 (91.6)
Yes	71 (1.4)	2 (2.2)	73 (1.4)
Missing	367 (7.0)	3 (3.3)	370 (7.0)
**Partner education**			
Less than 8 years	2405 (46.2)	39 (43.3)	2444 (46.1)
8 or more years	2245 (43.1)	37 (41.1)	2282 (43.1)
Missing*	556 (10.7)	14 (15.6)	570 (10.8)
**Partner drinks alcohol**			
No	4007 (77.0)	65 (72.2)	4072 (76.9)
Yes	749 (14.4)	16 (17.8)	765 (14.4)
Missing*	80 (1.5)	0 (0.0)	80 (1.5)
**Wealth tertiles**	370 (7.1)	9 (10.0)	379 (7.2)
Poorest	1731 (33.3%)	26 (28.9)	1757 (33.2)
Middle	1738 (33.4)	36 (40.0)	1774 (33.5)
Richest	1737 (33.4)	28 (31.1)	1765 (33.3)

*Data about this variable was not captured among some participants.

Among the enrolled women, only 3 out of 5296 did not have any antenatal visits and 84.1% gave birth in a health facility (Table 2). Of the women who had health facility deliveries, 69.8% delivered either from either a health centre IV or III and 14.3% delivered from a private clinic, health centre II, district hospital or regional referral hospital. In previous pregnancies, 18.4% had at least one stillbirth or miscarriage. In the current pregnancy, 92.1% had vaginal births, giving a caesarean section rate of 7.9%. There were no assisted vaginal births. Among the recruited women, 38.0% had complications in pregnancy, out of whom 24.6% had malaria. Few participants (1.7%) reported living with HIV.

**Table 2.** Clinical characteristics of study participants (*N* = 5296).

	Term live births	Term stillbirths	Total
	N = 5206 n (%)	N = 90 n (%)	N = 5296 n (%)
**Malaria in pregnancy**			
No	3928 (75.5)	66 (73.3)	3994 (75.4)
Yes	1278 (24.5)	24 (26.7)	1302 (24.6)
**Other fever**			
No	5017 (96.4)	88 (97.8)	5105 (96.4)
Yes	189 (3.6)	2 (2.2)	191 (3.6)
**Maternal HIV status**			
Negative	4683 (90.0)	87 (96.7)	4770 (90.1)
Positive	90 (1.7)	2 (2.2)	92 (1.7)
Missing*	433 (8.3)	1 (1.1)	434 (8.2)
**Child sex**			
Boy	2618 (50.3)	53 (58.9)	2671 (50.4)
Girl	2588 (49.7)	37 (41.1)	2625 (49.6)
**Antenatal care visits**			
Less than 4	3009 (57.8)	45 (50.0)	3054 (57.7)
More than 4	2197 (42.2)	45 (50.0)	2242 (42.3)
**Type of delivery**			
Vaginal birth	4807 (92.3)	69 (76.7)	4876 (92.1)
Caeserean section	399 (7.7)	21 (23.3)	420 (7.9)
**Birth place**			
Home birth	828 (15.9)	12 (13.3)	840 (15.9)
Health facility birth	4378 (84.1)	78 (86.7)	4456 (84.1)
**Pregnancy loss**			
No	4258 (81.8)	66 (73.3)	4324 (81.6)
Yes	948 (18.2)	24 (26.7)	972 (18.4)
**Number of previous births**			
No previous births	1595 (30.6)	28 (31.1)	1623 (30.6)
1–4 previous births	2813 (54.0)	40 (44.4)	2853 (53.9)
5 or more previous births	791 (15.2)	22 (24.4)	813 (15.4)
Missing*	7 (0.1)	0 (0.0)	7 (0.1)

*Data about this variable was not captured among some participants.

### Incidence of term stillbirth

There were 101 term stillbirths out of 5496 births resulting in an incidence of term stillbirth of 18.4 per 1000 births (95% CI 14.8 to 22.9). We obtained similar results when we used the mid-period population as our denominator (average of population at risk at enrolment and population at outcome assessment). Of the total stillbirths, 60.4% (61/101) were intrapartum stillbirths and 39.6% (40/101) were antepartum stillbirths.

### Underlying causes of term stillbirths

The most common underlying causes attributed to the term stillbirths were prolonged or obstructed labour (31.7%), malaria (19.8%), nuchal cord or cord prolapse (11.9%), and maternal infections (chorioamnionitis, urinary tract infection and other infections (11.9%)). We were unable to ascertain the underlying cause in 8.1% of the term stillbirths (Table 3).

**Table 3.** Underlying causes of stillbirths (*N* = 101).

Cause	Frequency	Percentage
Prolonged/obstructed labour	32	31.7
Malaria	20	19.8
Cord accidents	12	11.9
Cause not determined	9	8.9
Chorioamnionitis	8	7.9
Other	7	6.9
Antepartum haemorrhage	5	5.0
Congenital abnormalities	4	4.0
Other infections	4	4.0

### Factors associated with term stillbirths

Women who had caesarean births, a history of intimate partner violence and maternal age above 35 years were more likely to have term stillbirths (Table 4).

**Table 4.** Multivariable analysis of risk factors for term stillbirths (*N* = 5296).

Variable	Risk ratio (RR) [95% CI]	P value	Adjusted RR [95% CI]	P value
**Maternal age**				
35 years or less	1		1	
More than 35 years	2.3 [1.32, 4.02]	0.003	**2.19 [1.24, 3.87]**	**0.007**
**Child sex**				
Male	1		1	
Female	0.71 [0.52, 0.98]	0.040	0.73 [0.49, 1.11]	0.143
**Maternal education**				
Less than 8 years	1		1	
8 or more years	1.36 [0.95, 1.94]	0.095	1.34 [0.87, 2.08]	0.184
**Maternal alcohol consumption**				
No	1		1	
Yes	0.84 [0.40, 1.77]	0.654	0.78 [0.38, 1.63]	0.516
**Intimate partner violence**				
No	1		1	
Yes	1.76 [1.15, 2.68]	0.009	**1.77 [1.11, 2.82]**	**0.016**
**Place of birth**				
Home	1		1	
Health facility birth	1.23 [0.67, 2.26]	0.495	0.95 [0.51, 1.78]	0.878
**Mode of delivery**				
Vaginal birth	1		1	
Caeserean section	3.50 [2.10, 5.85]	<0.001	**3.27 [2.00, 5.36]**	**<0.001**
**Malaria in pregnancy**				
No	1		1	
Yes	1.08 [0.71, 1.65]	0.705	0.97 [0.60, 1.56]	0.889
**Pregancy loss**				
No	1		1	
Yes	1.62 [1.04, 2.52]	0.033	1.41 [0.88, 2.25]	0.149
**Wealth tertile**				
Poorest	1		1	
Middle	1.38 [0.88, 2.18]	0.165	1.31 [0.79, 2.16]	0.297
Richest	1.13 [0.69, 1.84]	0.631	0.93 [0.53, 1.63]	0.793
**Antenatal visits**				
Less than 4	1		1	
4 or more	1.35 [0.85, 2.13]	0.207	1.23 [0.81, 1.88]	0.326

## Discussion

The incidence of term stillbirth in our study was high (18.4 per 1000 total births). This incidence is higher than term stillbirth incidences in high-income countries (HICs), where a lower incidence of term stillbirth of between 0.41 and 0.48 per 1000 pregnancies have been reported [29,30]. This is likely due to better antenatal and intrapartum care in the HICs. Our findings, despite not including preterm stillbirths that occurred between 28 and 36 weeks of gestation, are similar to Nakamya et al. [31] who conducted a secondary analysis of national health registry of health facility births in Uganda and found that the mean stillbirth rate (term and preterm stillbirths) in Uganda between 2014 and 2020 was 20 per 1000 total births. The findings of our study may therefore be more representative of the population incidence of term stillbirth in Eastern Uganda because it was a community-based study that captured both health facilities and home births. WHO estimates that Uganda’s current stillbirth rate is 15.1 per 1000 total births [15], lower than observed in our study. The higher term stillbirth incidence in our study indicates that Uganda will have to accelerate its efforts to achieve the WHO target for stillbirth rate, of 12 or less per 1000 total births, by 2030 [32].

The most common attributed causes of stillbirths in our study were intrapartum related complications and maternal infections. This finding is consistent with reports from other countries in sub-Saharan Africa and South-East Asia that reported complications of labour and birth to account for more than half of the intrapartum stillbirths in sub-Saharan Africa [33].

Intrapartum stillbirths are a sensitive indicator of the timeliness and quality of intrapartum care [2]. Our study found that it was more common to have intrapartum term stillbirths than antepartum term stillbirth. Contrary to our findings, most stillbirths in HICs are antepartum. According to a report by the United Nations inter-agency group for child mortality, 6% of stillbirths in HICs are intrapartum, and 94% are antepartum.

Our study found that intimate partner violence was a risk factor for term stillbirth. This finding is similar to findings in the previous research in Ethiopia that found intimate partner violence to be associated with stillbirth [34]. A case–control study in Pakistan also found that pregnant women were four times more likely to have a stillbirth if they experienced intimate partner violence compared to those who did not [35].

We observed a higher risk of stillbirth in women above 35 years compared to the younger women. This finding is similar to results of other studies in both HICs and LICs that have consistently found advanced maternal age to be a risk factor for stillbirth [20,36–39]. Previous studies in Uganda have also found advanced maternal age to be a risk factor for stillbirths and neonatal deaths [20,40].

Our study also found caesarean section to be associated with a higher risk of term stillbirth compared to vaginal births. This finding is consistent with a registry-based study in East Africa that found that caesarean births are associated with adverse perinatal outcomes, such as stillbirths, pre-discharge neonatal deaths and maternal deaths [41]. However, this association can be due to reverse causality where the foetus may have already been at risk of a stillbirth before the caesarean birth due to the complication that made a surgical birth a necessity. It is also possible that this finding may be due to residual confounding by factors, such as foetal distress, antepartum haemorrhage and others which are usually indications for caesarean section but are also potential risk factors for stillbirths. We did not have data on these factors to include in the model for multivariate analysis. The caesarean section rate in our study was 7.9%, and there were no assisted vaginal births. This caesarean section rate is lower than the minimum recommended by the WHO of 10% [42] reflecting an unmet need.

Our study had several limitations. We could not accurately assess the gestational age of the women at enrolment or at birth because almost no mother had undergone an early ultrasound scan. The classification of stillbirth into term and preterm was based on the woman’s report of the last normal menstrual period before conception. This may have led to non-differential misclassification bias. Almost all term stillbirths had no record of the birthweight despite most having given birth from a health facility. This is because health workers in the health facilities in the study area rarely weigh babies who are stillborn.

We determined the timing of stillbirths based on the health records at the health facility or the discharge summary the women were given after birth and verbal report from the mother. Presence or absence of maceration was also used. These methods may have led to nondifferential misclassification. The determination of the occurrence of maternal illness during pregnancy, such as malaria, was based on maternal verbal reports that the health worker made a diagnosis. How the health worker confirmed the diagnosis of malaria, especially during the period of pregnancy before the woman was enrolled in the study, was not clear and this may also have led to a non-differential misclassification bias. The true burden of malaria, urinary tract infection and other infections may have been higher than the reported figures because some of the women who had fever may have had some of these infections but the diagnosis was not confirmed by the health worker.

The causes of stillbirths for some of the participants in our study are based on verbal autopsies and need to be interpreted with caution. A larger community study involving several regions in Uganda could assess the countrywide burden and risk factors for term stillbirths.

## Conclusion

Eastern Uganda has a high incidence of term stillbirths with more than half occurring during labour. Based on our findings, efforts are needed to improve the quality of intrapartum care through effective labour monitoring, timely decision-making, effective referral mechanisms and readily available caesarean section delivery when needed, among others. Efforts are also needed to prevent intimate partner violence. Health workers should be supported to consistently do clinical assessments, including the birthweight of stillbirths to ensure better documentation of the burden of stillbirths, its timing and causes in this setting.

## Data Availability

The data supporting the results presented in this manuscript are available from the corresponding author on reasonable request.
